# Supplementary material for: Nucleosome spacing can fine-tune higher-order chromatin assembly
Source: Nat Commun. 2025 Jul 9;16:6315. doi: 10.1038/s41467-025-61482-x (PMC12238351; doi:10.1038/s41467-025-61482-x)
Supplement: Supplementary file 1 — Supplementary Information [file 41467_2025_61482_MOESM1_ESM.pdf]

Supplementary Fig. 1

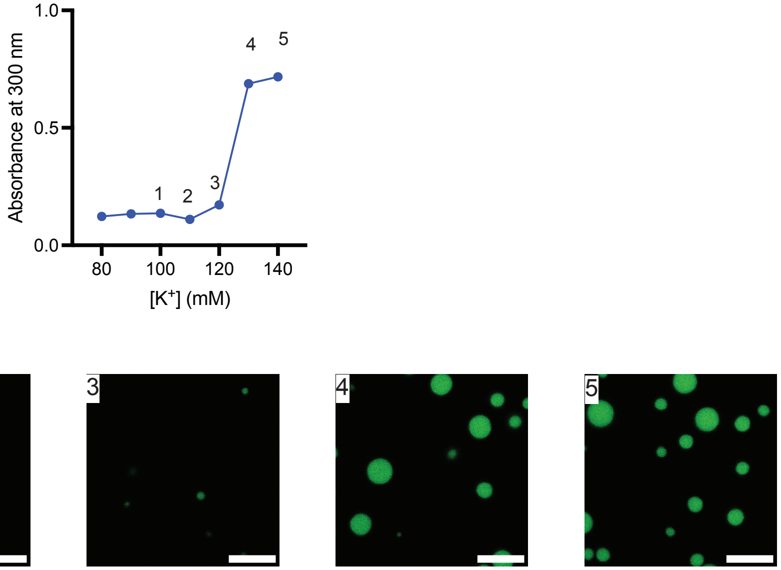

No phase separation
Baseline Absorbance

No phase separation
Baseline Absorbance

Just beyond the threshold
Increase in Absorbance

Beyond the threshold
High Absorbance

Beyond the threshold
High Absorbance

Supplementary Fig. 1. Turbidity assay correlate with microscopy.

Top, Turbidity assay. Each data point represents absorbance measured at 300 nm for the chromatin array in the K^+ concentration indicated. **Bottom**, fluorescence confocal microscopy images of the chromatin array. The numbered data points (1-5) for both the turbidity assay and the microscopy represent the same buffer condition (K^+ concentration). Scale bar: 10 µm.

Supplementary Fig. 2

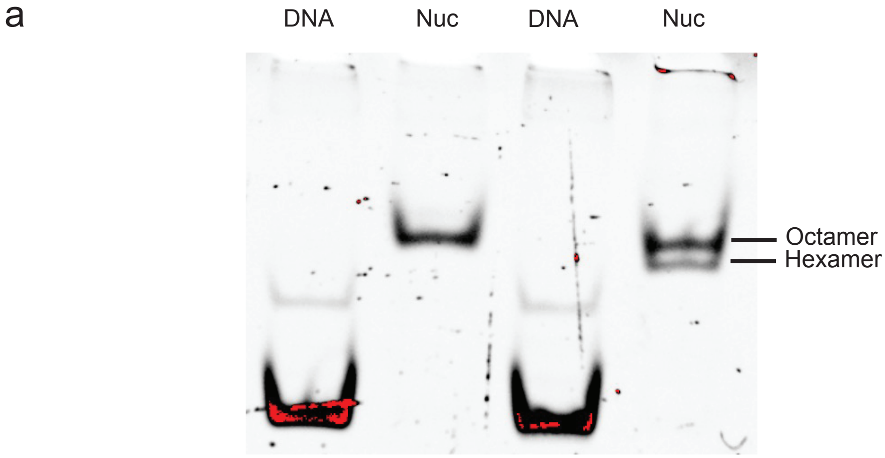

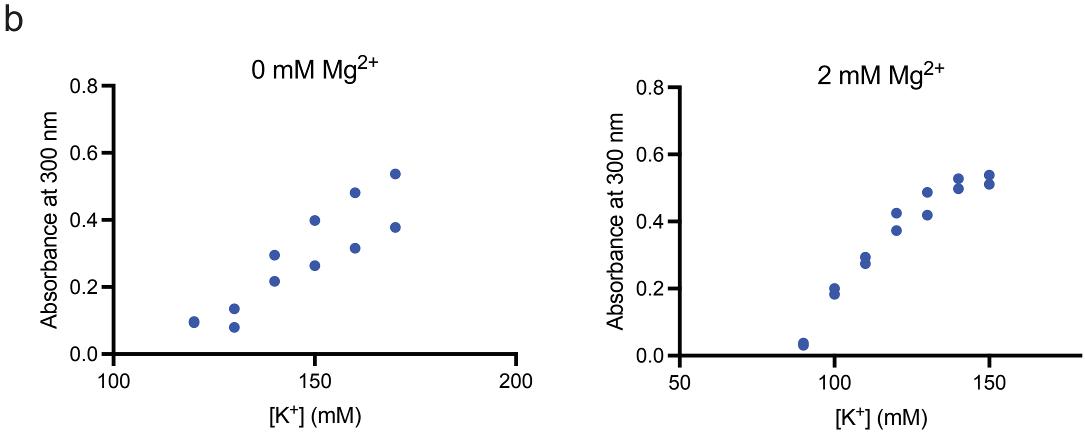

Supplementary Fig. 2. Hexamer-containing 30 bp chromatin arrays have a higher propensity to phase separate.

a, Electrophoretic mobility shift assay (EMSA) of digested DNA or chromatin arrays. Lane 2 shows fully assembled chromatin arrays post digestion to mononucleosomes. Lane 4 shows hexamer containing chromatin array post digestion to mononucleosomes. **b**, Turbidity assay for hexamer containing 30 bp chromatin arrays at 0 mM Mg^{2+} (left) or 2 mM Mg^{2+} (right) at K^{+} concentrations indicated, with two replicates each.

Supplementary Fig. 3

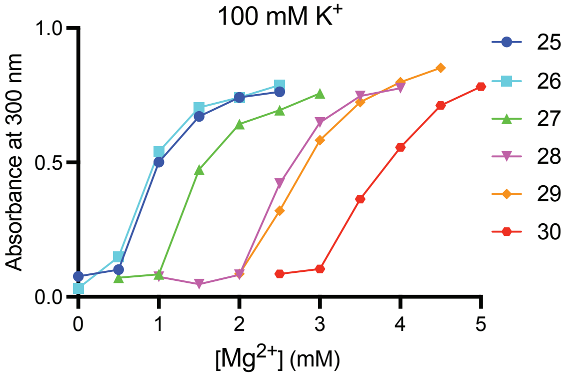

Supplementary Fig. 3. All tested chromatin arrays phase separate at high Mg^{2+} concentrations.

Turbidity assay for arrays with linker DNA lengths from 25 bp to 30 bp. Each data point represents the absorbance measured at 300 nm for arrays in 100 mM K^+ and Mg^{2+} concentration indicated.

Supplementary Fig. 4

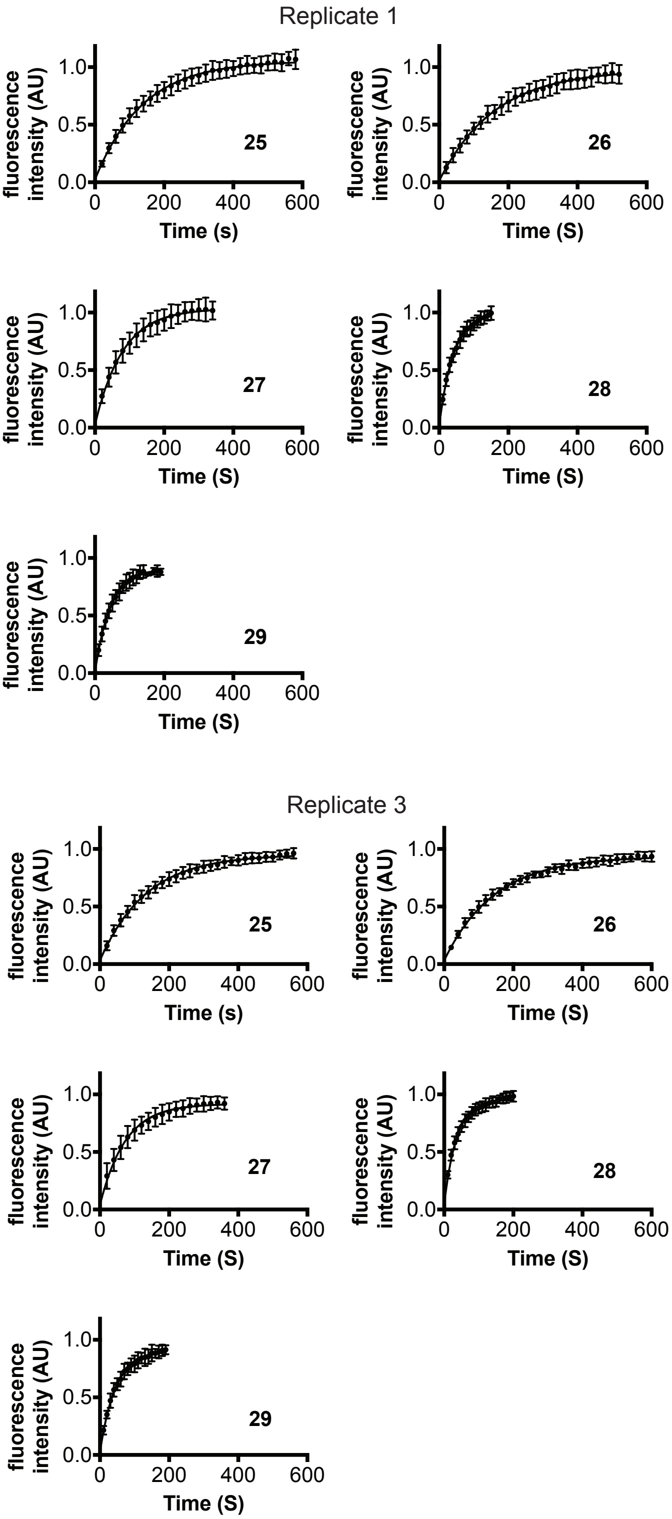

Supplementary Fig. 4. Fluorescence recovery after photobleaching (FRAP) of chromatin arrays without magnesium in buffer. Fluorescence recovery over time of a central bleached region in 25 bp to 29 bp chromatin condensates in 180 mM K^+ and 0 mM Mg^{2+}. Three sets of replicates are shown. Data are represented as normalized mean fluorescence intensity ± s.d.

Supplementary Fig. 5

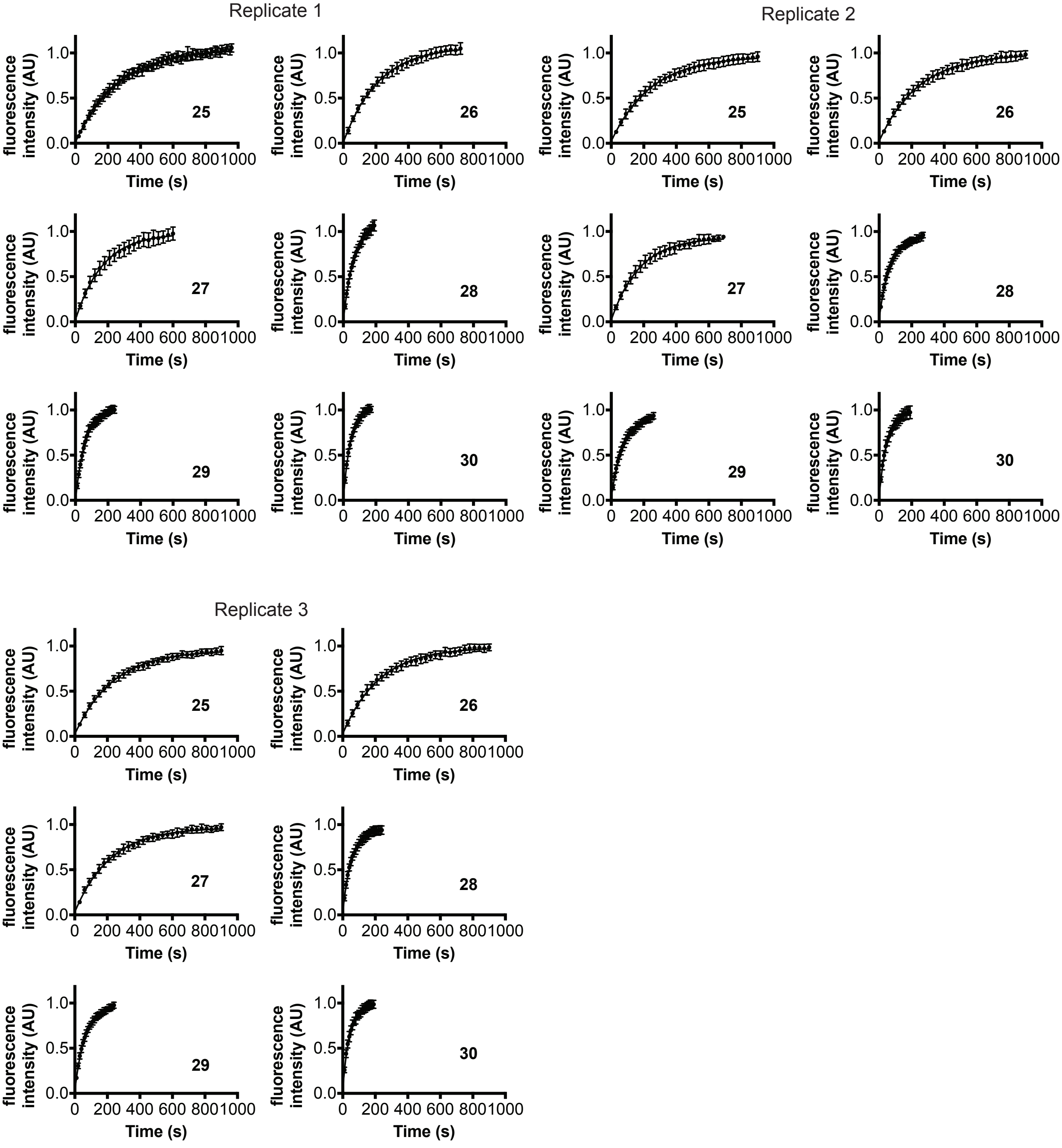

Supplementary Fig. 5: Fluorescence recovery after photobleaching (FRAP) of chromatin arrays in magnesium buffer. Fluorescence recovery over time of a central bleached region in 25 bp to 30 bp chromatin condensates in 180 mM K^+ and 3 mM Mg^{2+}. Three sets of replicates are shown. Data are represented as normalized mean fluorescence intensity ± s.d.

Supplementary Fig. 6

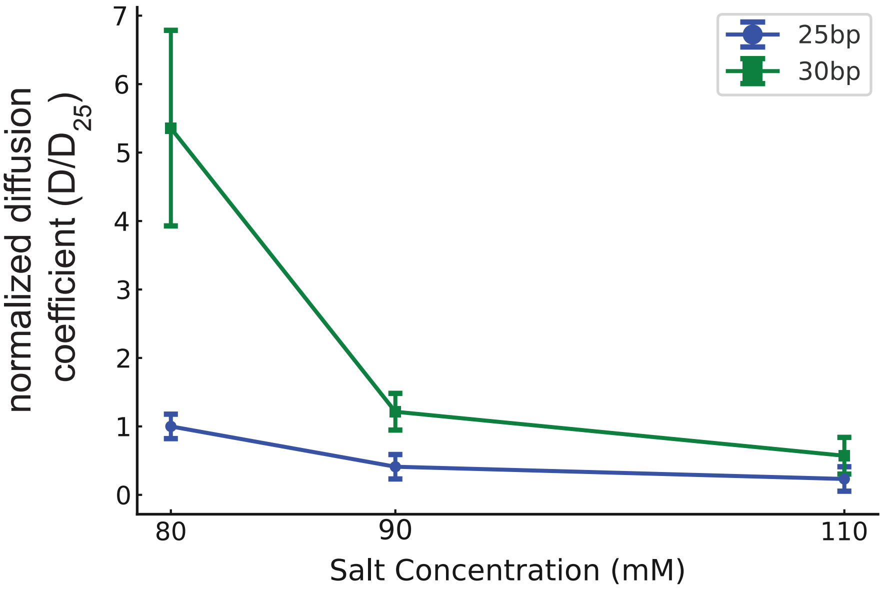

Supplementary Fig. 6. Diffusion coefficients at different salt concentrations. Simulated diffusion coefficient, for the indicated linker lengths, at several concentrations of monovalent salt (NaCl). Across the salt regime considered, 25 bp arrays (blue) have a lower diffusion coefficient than the 30 bp arrays (orange). Data are represented as mean ± s.d. Error bars were computed using block averaging over the entire simulation trajectory, consisting of n = 700 points.

Supplementary Fig. 7

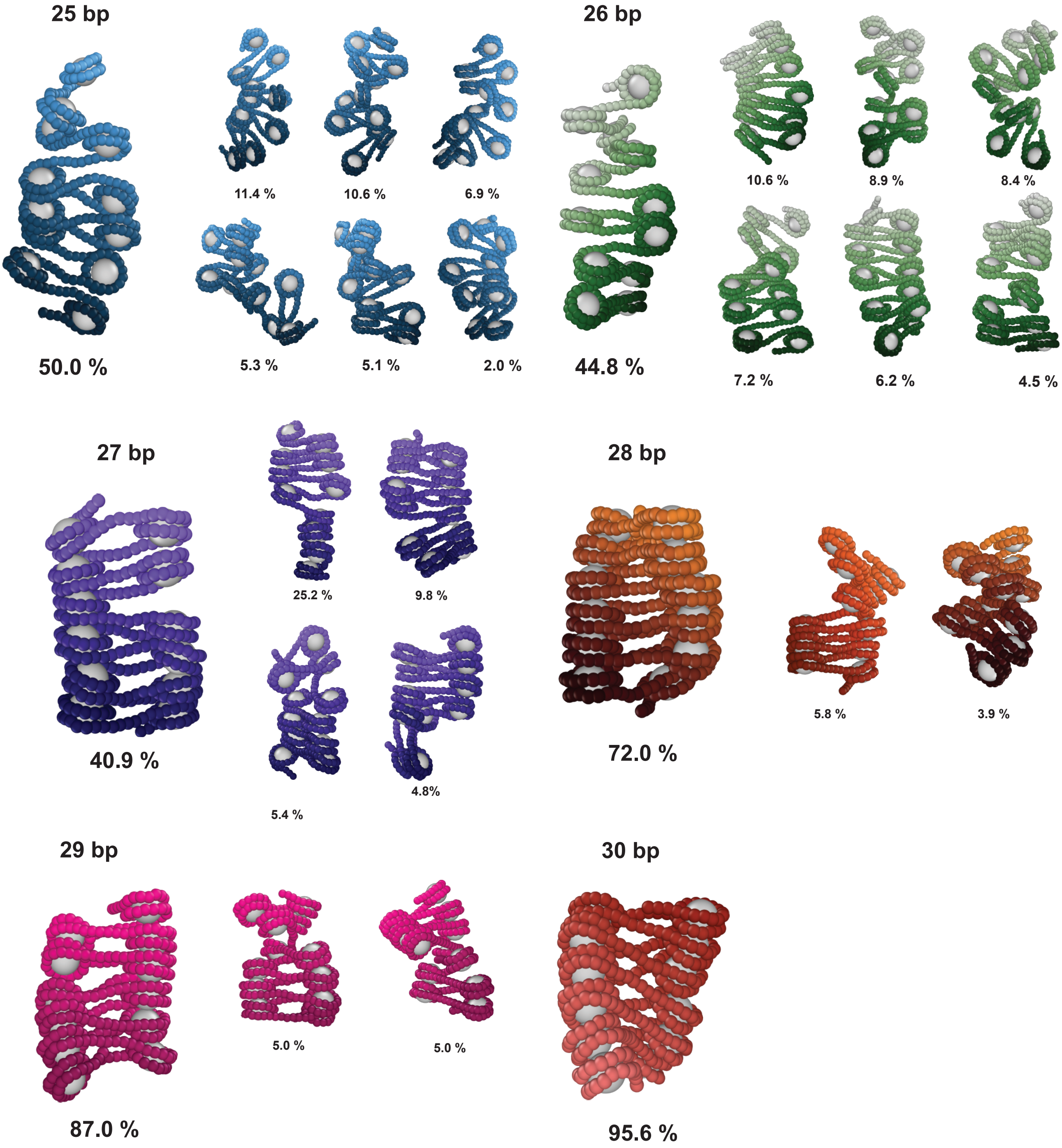

Supplementary Fig. 7. Extended representative simulation snapshots. Simulation snapshots of the most common structures found in the bulk of the condensate at 1.2 normalized salt conditions. The distance-RMSD of all fibers in the bulk was computed, in order to then cluster together via k-means the most similar fibers of each linker length. Here, the largest clusters, representing over 90% of the data, are depicted for each linker length: 25 bp (blue), 26 bp (green), 27 bp (purple), 28 bp (orange), 29 bp (pink) and 30 bp (red); with their corresponding percentages.

Supplementary Fig. 8

15 bp

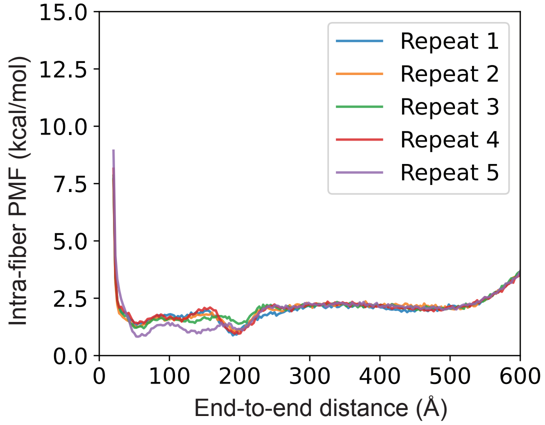

20 bp

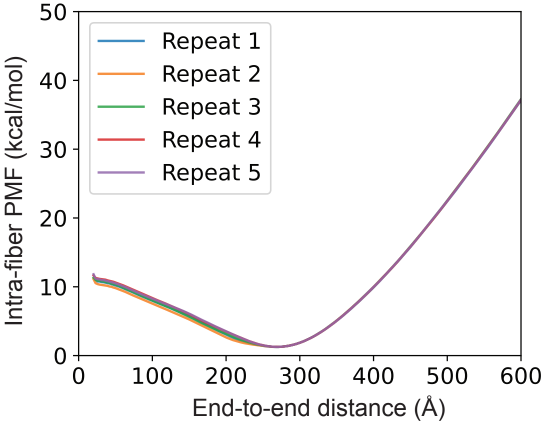

25 bp

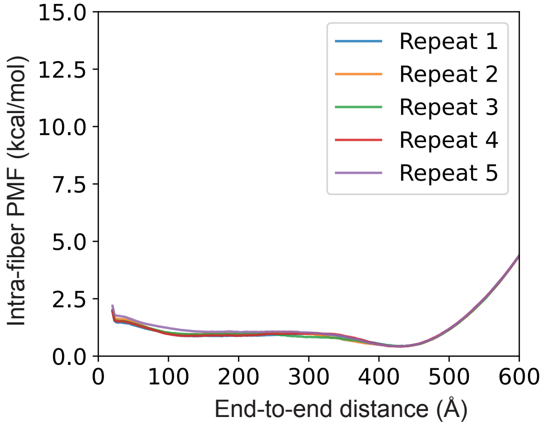

30 bp

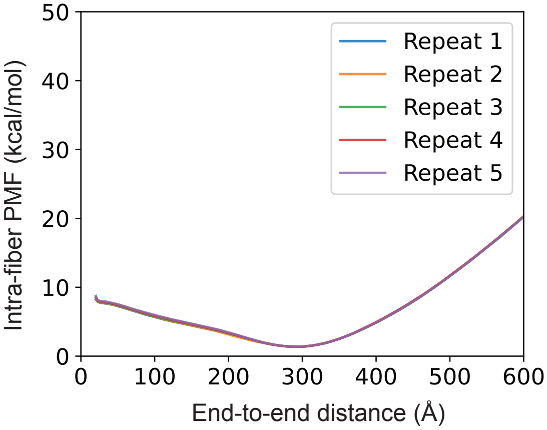

35 bp

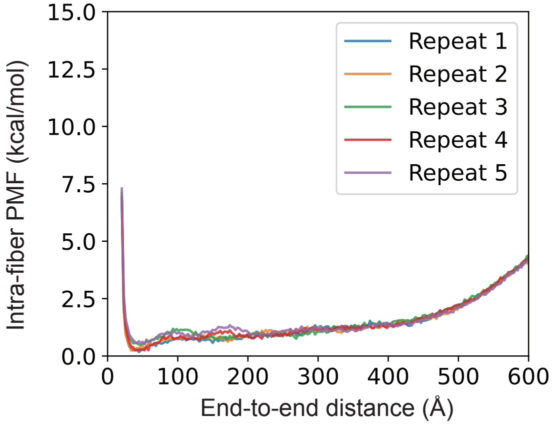

Supplementary Fig. 8. End-to-end extension of individual chromatin fibers. Related to Fig. 3d, where each replicate of end-to-end extension of arrays of indicated linker length is shown.

Supplementary Fig. 9

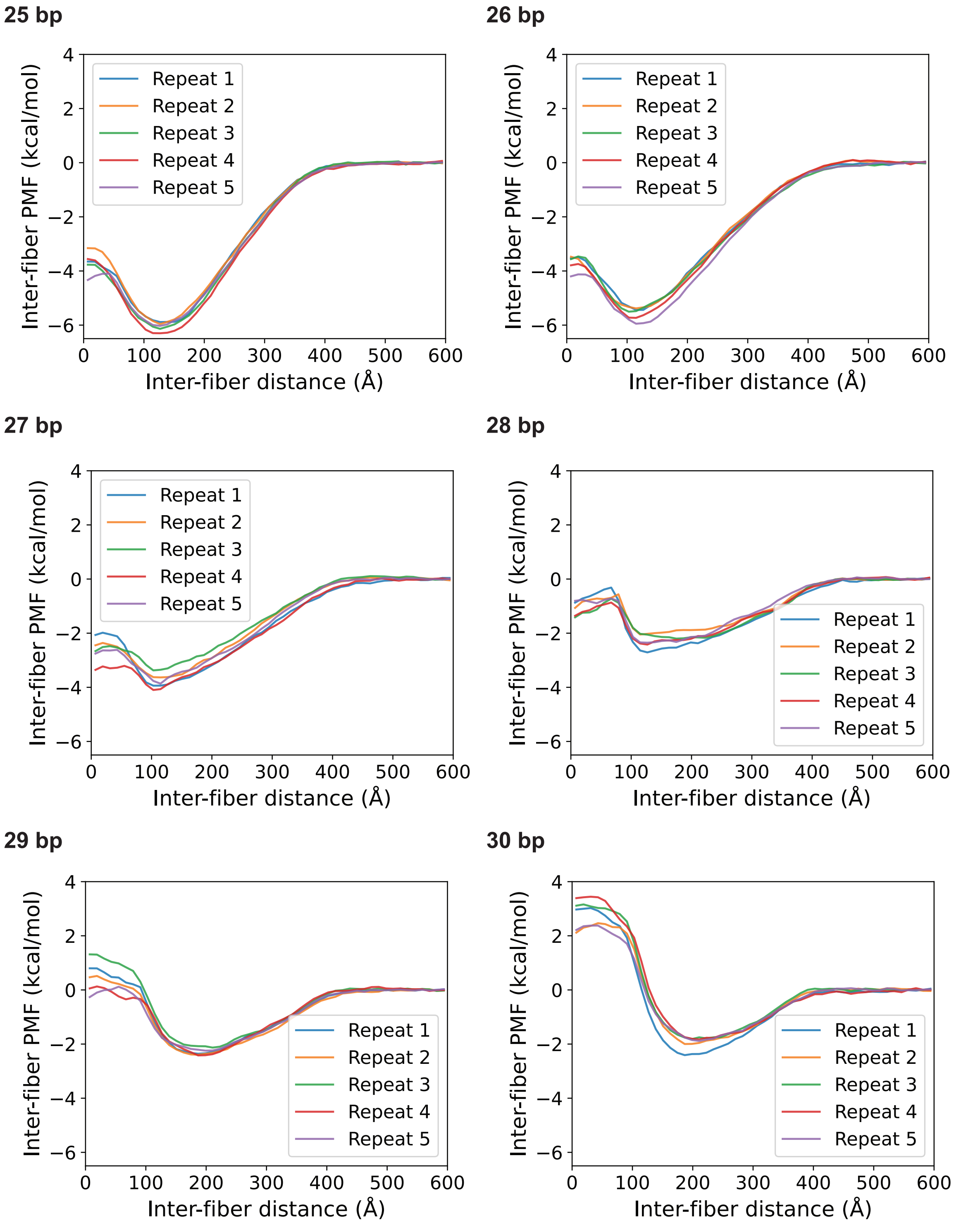

Supplementary Fig. 9. PMFs of pairs of chromatin fibers. Related to Fig. 4c, where each replicate of PMFs of pairs of chromatin fibers of indicated linker length is shown.

Supplementary Fig. 10

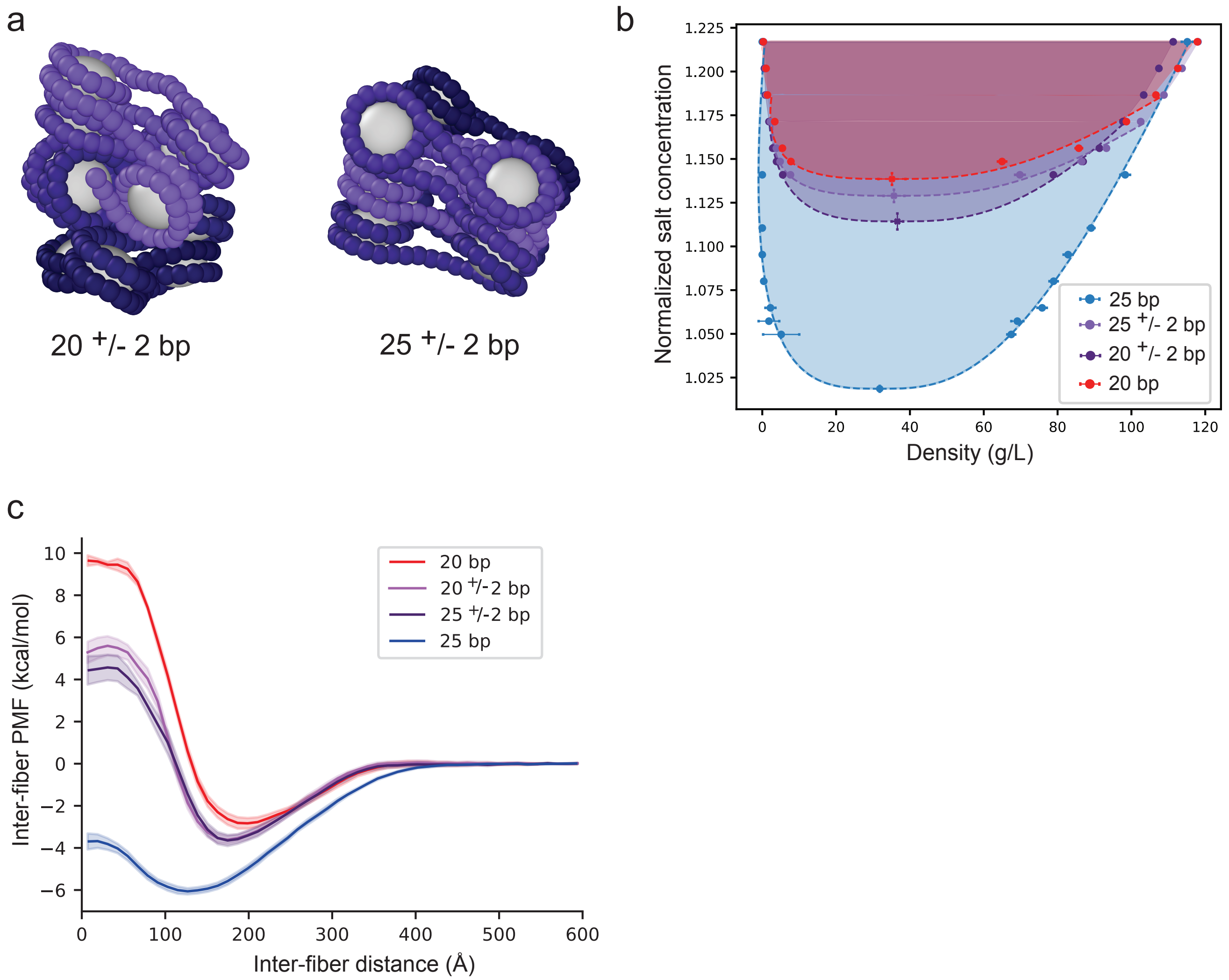

Supplementary Fig. 10. Simulated phase behavior of chromatin arrays with irregular linker DNA lengths. a, Representative structures of 20 ± 2 bp arrays (left) and 25 ± 2 bp arrays (right). **b,** Simulation coexistence curves for arrays with indicated regular and irregular linker lengths. Circular points represent chromatin density in the dilute phase (left branch) and dense phase (right branch) at a given monovalent salt concentration. Data are represented as mean ± s.d. (error bars are smaller than the symbols in most cases). Critical points are calculated by fitting the data to equations (7) and (8), and the error bars are ± error from the least-squares fitting procedure. The dashed lines represent coexistence curves and are obtained by fitting the data to equations (7) and (8). **c,** PMF calculated for interactions between pairs of chromatin arrays with the indicated linker length at 150 mM monovalent salt. Distance indicates inter-fiber distance between the geometric centers of the fibers. Computed using umbrella sampling. $n = 5$ independent repeats. Solid curves represent the mean, and the shading represents the standard deviation.

Supplementary Fig. 11

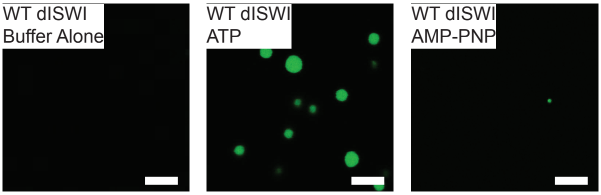

Supplementary Fig. 11. WT ISWI induces 30 bp LLPS. Remodeling 30 bp nucleosome arrays to 35 bp arrays by WT ISWI. Representative images of remodeling reactions with conditions indicated after a 6-hour incubation. n = 3 independent replicates. Scale bar: 10 μm.

Supplementary Fig. 12

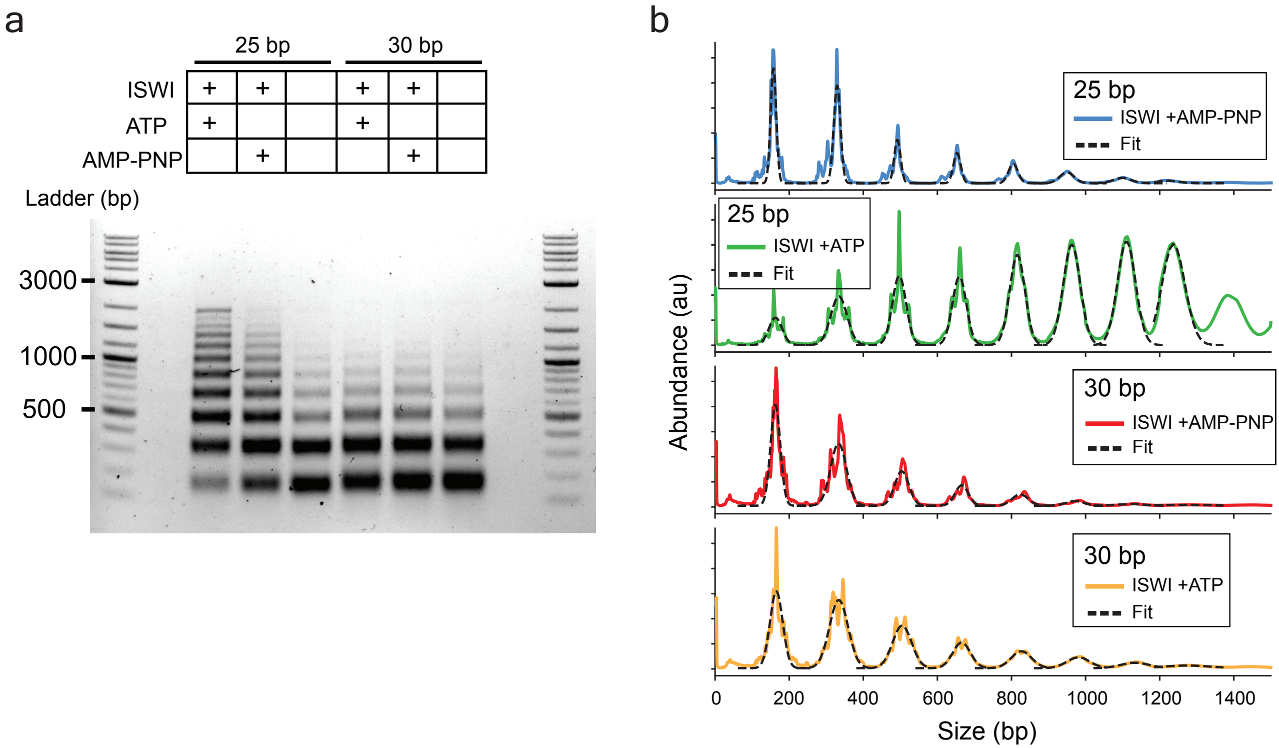

Supplementary Fig. 12. ISWI increases linker lengths. a, Agarose gel of MNase digested nucleosome arrays (25 bp and 30 bp) with indicated reaction conditions (ISWI-2RA, ATP, AMP-PNP). **b**, Bioanalyzer result traces for MNase digested nucleosome arrays (25 bp and 30 bp) with indicated reaction conditions with overlays for peak fitting.
