# Supplementary material for: Nucleosome spacing can fine-tune higher-order chromatin assembly
Source: Nat Commun. 2025 Jul 9;16:6315. doi: 10.1038/s41467-025-61482-x (PMC12238351; doi:10.1038/s41467-025-61482-x)
Supplement: Supplementary file 3 — Description of Additional Supplementary Files [file 41467_2025_61482_MOESM3_ESM.pdf]

Description of Additional Supplementary Files

File Name: Supplementary Data 1
Description: Contains DNA sequences of arrays used in this study.

File Name: Supplementary Data 2
Description: Contains interpolated thresholds and errors and fitted FRAP parameters.

File Name: Supplementary Data 3
Description: Contains the analysis code and the resulting data for Fig. 6 and Supplementary Fig.12.
