# Supplementary material for: Nucleosome spacing can fine-tune higher-order chromatin assembly
Source: Nat Commun. 2025 Jul 9;16:6315. doi: 10.1038/s41467-025-61482-x (PMC12238351; doi:10.1038/s41467-025-61482-x)
Supplement: Supplementary file 7 — Reporting Summary [file 41467_2025_61482_MOESM7_ESM.pdf]

nature portfolio

Corresponding author(s): Michael K. Rosen, Rosana Collepardo-Guevara

Last updated by author(s): May 22, 2025

Reporting Summary

Nature Portfolio wishes to improve the reproducibility of the work that we publish. This form provides structure for consistency and transparency in reporting. For further information on Nature Portfolio policies, see our Editorial Policies and the Editorial Policy Checklist.

Statistics

For all statistical analyses, confirm that the following items are present in the figure legend, table legend, main text, or Methods section.

n/a	Confirmed	
☐	☒	The exact sample size (*n*) for each experimental group/condition, given as a discrete number and unit of measurement
☐	☒	A statement on whether measurements were taken from distinct samples or whether the same sample was measured repeatedly
☒	☐	The statistical test(s) used AND whether they are one- or two-sided<br>*Only common tests should be described solely by name; describe more complex techniques in the Methods section.*
☒	☐	A description of all covariates tested
☒	☐	A description of any assumptions or corrections, such as tests of normality and adjustment for multiple comparisons
☐	☒	A full description of the statistical parameters including central tendency (e.g. means) or other basic estimates (e.g. regression coefficient) AND variation (e.g. standard deviation) or associated estimates of uncertainty (e.g. confidence intervals)
☒	☐	For null hypothesis testing, the test statistic (e.g. *F*, *t*, *r*) with confidence intervals, effect sizes, degrees of freedom and *P* value noted<br>*Give P values as exact values whenever suitable.*
☒	☐	For Bayesian analysis, information on the choice of priors and Markov chain Monte Carlo settings
☒	☐	For hierarchical and complex designs, identification of the appropriate level for tests and full reporting of outcomes
☒	☐	Estimates of effect sizes (e.g. Cohen's *d*, Pearson's *r*), indicating how they were calculated

Our web collection on statistics for biologists contains articles on many of the points above.

Software and code

Policy information about availability of computer code

Data collection	Molecular dynamics simulations were performed using modified LAMMPS on the Cambridge Service for Data Driven Discovery (CSD3) and ARCHER2 high-performance computing clusters. Source code can be found at https://github.com/CollepardoLab/NucleosomeLinker/ and https://doi.org/10.5281/zenodo.15351283.
Data analysis	FRAP data was fitted by Graphpad Prism 10 using one-phase association. Microscopy images were processed by ImageJ. Remodeler fragment analysis was performed on custom code provided in the Source Data file.<br>Simulation trajectories were analyzed using standard molecular dynamics tools (MDAnalysis, AmberTools) and in-house Python scripts. Analyses focused on extracting structural and dynamic properties relevant to phase behaviour, as described in the Methods section. Source code can be found at https://github.com/CollepardoLab/NucleosomeLinker/ and https://doi.org/10.5281/zenodo.15351283.

For manuscripts utilizing custom algorithms or software that are central to the research but not yet described in published literature, software must be made available to editors and reviewers. We strongly encourage code deposition in a community repository (e.g. GitHub). See the Nature Portfolio guidelines for submitting code & software for further information.

Data

Policy information about availability of data

All manuscripts must include a data availability statement. This statement should provide the following information, where applicable:

- Accession codes, unique identifiers, or web links for publicly available datasets
- A description of any restrictions on data availability
- For clinical datasets or third party data, please ensure that the statement adheres to our policy

The in vitro data generated are provide in the Source Data file. The computational datasets generated during the current study are stored on https://doi.org/10.6084/m9.figshare.28672877.

Research involving human participants, their data, or biological material

Policy information about studies with human participants or human data. See also policy information about sex, gender (identity/presentation), and sexual orientation and race, ethnicity and racism.

Reporting on sex and gender	N/A
Reporting on race, ethnicity, or other socially relevant groupings	N/A
Population characteristics	N/A
Recruitment	N/A
Ethics oversight	N/A

Note that full information on the approval of the study protocol must also be provided in the manuscript.

Field-specific reporting

Please select the one below that is the best fit for your research. If you are not sure, read the appropriate sections before making your selection.

☒ Life sciences ☐ Behavioural & social sciences ☐ Ecological, evolutionary & environmental sciences

For a reference copy of the document with all sections, see nature.com/documents/nr-reporting-summary-flat.pdf

Life sciences study design

All studies must disclose on these points even when the disclosure is negative.

Sample size	Samples sizes are indicated in the main text for in vitro assays and were not predetermined. The in vitro results were highly reproducible across many biological and technical replicates. Computational simulations were run for long timescales (over 100 million timesteps of simulation time), with data recorded every 10000 timestep, to ensure convergence, with extensive thermalization prior to data collection.
Data exclusions	For FRAP analyses, droplets were excluded if fusion events occurred during the recovery or if the bleached spot was near the droplet periphery. The first 20 million timesteps of simulation were discarded to ensure proper thermalization and convergence of computational simulations. No other data were excluded from the simulation trajectories before analysis.
Replication	The in vitro results were all successfully reproduced. All key computational findings were reproduced across multiple independent simulation replicates.
Randomization	Initial configurations for simulations were generated using randomized starting positions and velocities to ensure unbiased sampling. Each system was simulated on single molecule via replica exchange before condensate production runs to enhance sampling and promote equilibration.
Blinding	The investigators were not blinded to allocation during experiments and outcome assessment, as our experiments provided quantitative and objective measurements.

Reporting for specific materials, systems and methods

We require information from authors about some types of materials, experimental systems and methods used in many studies. Here, indicate whether each material, system or method listed is relevant to your study. If you are not sure if a list item applies to your research, read the appropriate section before selecting a response.

Materials & experimental systems

n/a	Involved in the study
☒	☐ Antibodies
☒	☐ Eukaryotic cell lines
☒	☐ Palaeontology and archaeology
☒	☐ Animals and other organisms
☒	☐ Clinical data
☒	☐ Dual use research of concern
☒	☐ Plants

Methods

n/a	Involved in the study
☒	☐ ChIP-seq
☒	☐ Flow cytometry
☒	☐ MRI-based neuroimaging

Plants

Seed stocks	*Report on the source of all seed stocks or other plant material used. If applicable, state the seed stock centre and catalogue number. If plant specimens were collected from the field, describe the collection location, date and sampling procedures.*
Novel plant genotypes	*Describe the methods by which all novel plant genotypes were produced. This includes those generated by transgenic approaches, gene editing, chemical/radiation-based mutagenesis and hybridization. For transgenic lines, describe the transformation method, the number of independent lines analyzed and the generation upon which experiments were performed. For gene-edited lines, describe the editor used, the endogenous sequence targeted for editing, the targeting guide RNA sequence (if applicable) and how the editor was applied.*
Authentication	*Describe any authentication procedures for each seed stock used or novel genotype generated. Describe any experiments used to assess the effect of a mutation and, where applicable, how potential secondary effects (e.g. second site T-DNA insertions, mosiacism, off-target gene editing) were examined.*
